# Supplementary material for: Platelets favor the outgrowth of established metastases
Source: Nat Commun. 2024 May 13;15:3297. doi: 10.1038/s41467-024-47516-w (PMC11091169; doi:10.1038/s41467-024-47516-w)
Supplement: Supplementary file 1 — Supplementary Information [file 41467_2024_47516_MOESM1_ESM.pdf]

Supplementary information for :

Platelets favor the outgrowth of established metastases

Maria J. Garcia-Leon[1,2,3,4*#§], Cristina Liboni[1,2,3,4§], Vincent Mittelheisser[1,2,3,4§], Louis Bochler[1,2,3,4], Gautier Follain[1,2,3,4#], Clarisse Mouriaux[5], Ignacio Busnelli[1,2,3,4], Annabel Larnicol[1,2,3,4], Florent Colin[1,2,3,4], Marina Peralta[1,2,3,4], Naël Osmani[1,2,3,4], Valentin Gensbittel[1,2,3,4], Catherine Bourdon[5], Rafael Samaniego[6], Angélique Pichot[2,3,7], Nicodème Paul[2,3,7], Anne Molitor[2,3,7], Raphaël Carapito[2,3,7,8], Martine Jandrot-Perrus[9], Olivier Lefebvre[1,2,3,4], Pierre H. Mangin[5*] and Jacky G. Goetz[1,2,3,4*]

*** Correspondence:**

Maria J Garcia-Leon, mgarcialeon@domaintherapeutics.com

Pierre Mangin, Pierre.Mangin@efs.sante.fr

Jacky G. Goetz, jacky.goetz@inserm.fr

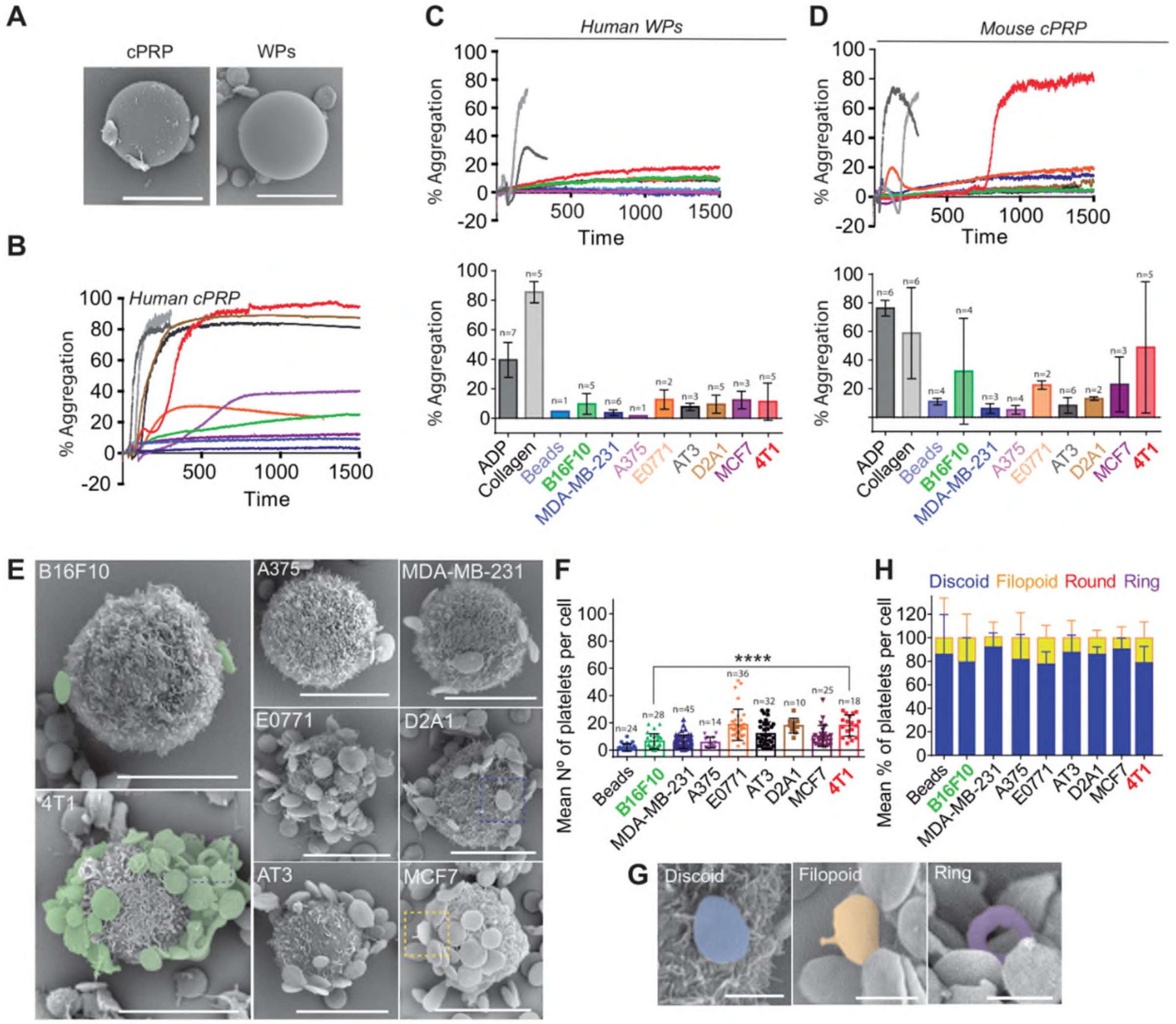

Supplementary Figure 1 (related to Figure 1). Garcia-Leon *et al.*

Supplementary Figure 1 *(related to Figure 1)*. SEM and TCIPA control and representative aggregation curves. (A) SEM images of PEG-treated 10µm polystyrene beads interacting with mouse platelets contained within mouse citrated platelet rich plasma (cPRP) or human washed platelets (WPs). Scale bar: 10µm**. (B)** Representative platelet aggregation curves obtained with human cPRP for each TCs line, with ADP and collagen as positive controls, while beads as negative control (1 experiment was performed). **(C, D)** Representative platelet aggregation curves (up) and platelet aggregation efficiency (%) (down) of the different TCs tested using human WPs (C) or mouse cPRP (D). The percentage of aggregation for each TCs (number of aggregation curves analyzed are indicated above the histogram) is represented as mean ± SD, 4 independent aggregation experiments were performed. **(E)** SEM images

of TCs interacting with human washed platelets (WPs). 4T1 and B16F10 cells are highlighted. Scale bar: 8µm**. (F)** Platelet binding profiles quantification graph of (E). Mean number of platelets per TCs (number of TCs used is indicated above the histogram) is represented as mean ± SD and analyzed using Kruskal-Wallis with false discovery rate post-test, $p<0.0001$, 4 independent experiments were performed. **(G)** SEM images of TC-bound platelets at different activation states: discoid (resting), filopodia (low activation) and ring-shaped (unknown function). Scale bar: 2µm**. (H)** Mean percentage of TC-bound platelets (number of beads n=16, numbers of B16F10 n=26, MDA-BM-231 n=42, A375 n=14, E0771 n=36, AT3 n=32, D2A1 n=10, MCF7 n=26, 4T1 n=18) and their respective activation states, mean ± SD are shown, 3 independent experiments were performed. Source data are provided as a Source Data file.

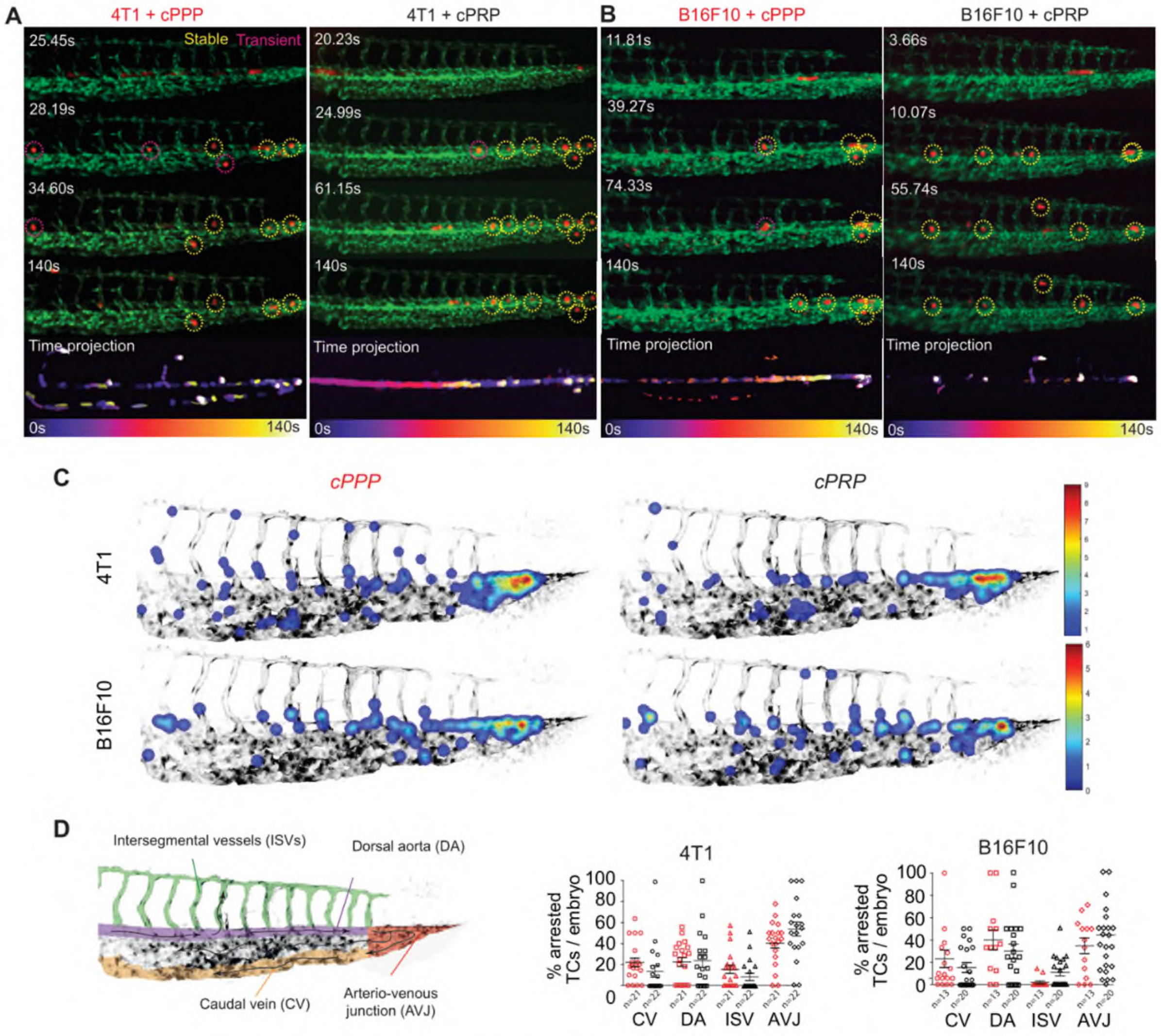

Supplementary Figure 2 (related to Figure 2). Garcia-Leon *et al.*

Supplementary Figure 2 *(related to Figure 2)*. TCs interaction with platelets enhances their intravascular adhesion to the endothelial wall in a cell type-specific manner (A, B) Live video microscopy time-frames and time projection of 4T1 (A) and B16F10 (B) CTCs circulation and arrest within the ZF caudal plexus with human cPRP (citrated Platelet-Rich Plasma) and the absence (cPPP, citrated Plasma-Poor Plasma). Yellow circles highlight 4T1 cells that circulate and arrest in a sole position during the time of the assay (stable arrest). Magenta circles highlight B16F10 cells that circulate and/or arrest briefly (transient arrest). 20 embryos were analyzed from 4 independent experiments. **(C)** Analysis by MatLab heat mapping of 4T1 and B16F10 CTCs arrest position in the presence (cPRP) or absence (cPPP) of human platelets at 5mpi. **(D)** On the left, areas of the ZF circulatory system are depicted. On the right, manual counting of CTCs number per ZF by vessel type (CV: caudal veins;

DA: dorsal aorta; ISV: intersegmental vessels; AVJ: arterio-venous junction) in 4T1 and B16F10 models respectively, in the presence (cPRP - black) or absence (cPPP - red) of human platelets. Mean percentage ± SD of arrested TCs per embryo (number of embryos indicated in the histogram) is represented and analyzed using two-sided t test, 4 independent experiments were performed. Source data are provided as a Source Data file

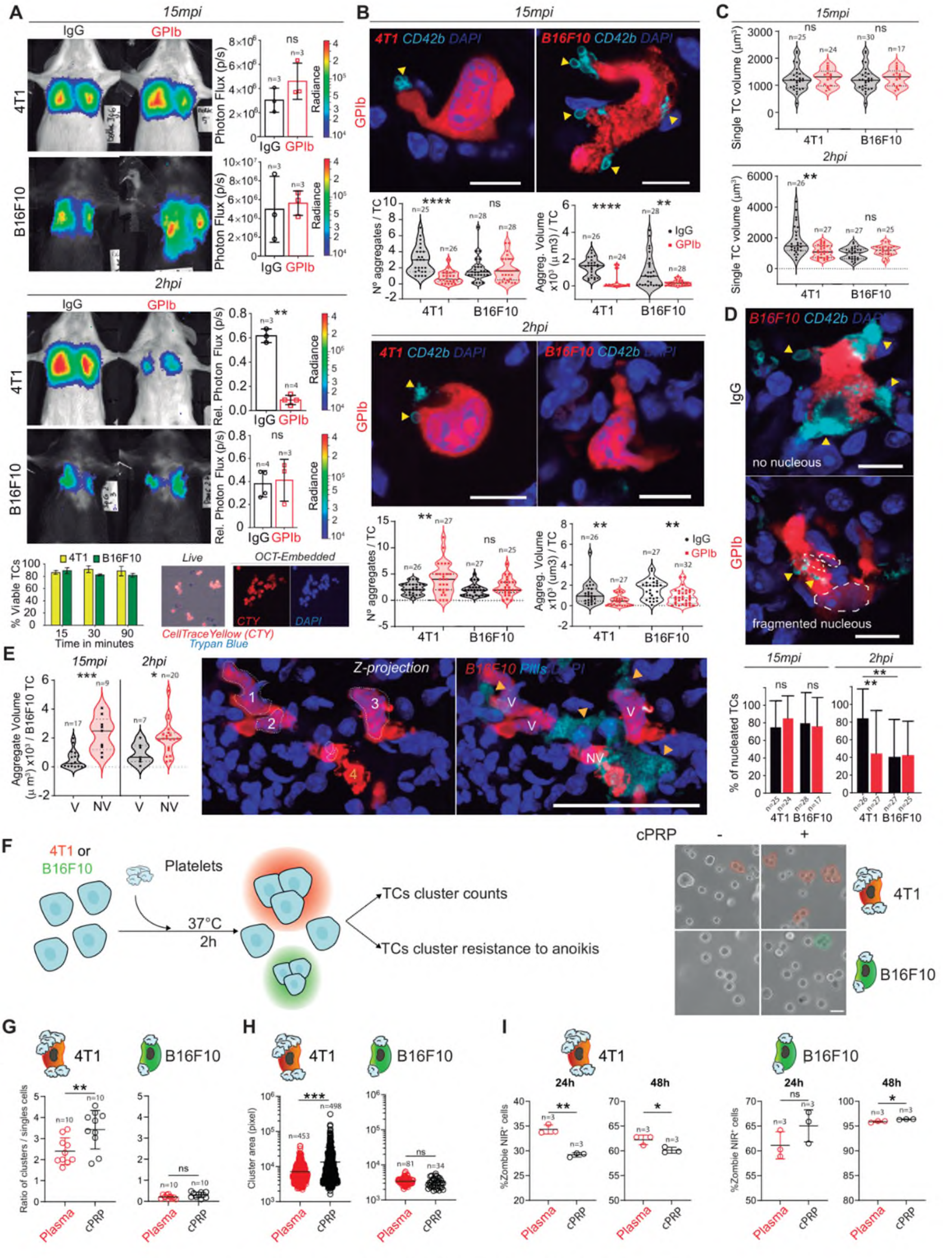

Supplementary Figure 3 (related to Figure 2). Garcia-Leon *et al.*

Supplementary Figure 3 *(related to Figure 2)*. TCs' active or passive interaction with platelets enhances their intravascular survival. (A) Representative images

and time point comparisons of total lung bioluminescence in normal versus TCP mice (number of mice per group is indicated above the histogram) in the 4T1 (up) and B16F10 (down) TCs models used for IF analyses of TC intravascular arrest at 15mpi (upper panel) and 2hpi (lower panel). Mean photon flux (p/s) (up) or mean relative photon flux (p/s) (down) ±SD is represented and analyzed using two-sided Mann Whitney test. Bottom left, relative *in vitro* viabilities of 4T1 and B16F10 cells up to 90min shown as mean ± SD and analyzed using two-way Anova with false discovery rate post-test (n=3 replicates per cell line, 1 experiment was performed). Bottom right, live and fluorescence imaging of and PFA-fixed / OCT-embedded 4T1 TCs labelled with CellTrace Yellow and DAPI. **(B)** Representative AMIRA segmentation images of single TCs arrested intravascularly in the mouse lungs 15mpi (upper panel) and 2hpi (lower panel). Scale: 10µm. Mean ± SD of the number of aggregates per TCs left (up, 4T1 p<0.0001; down, 4T1 p<0.0082) or aggregation volume per TCs right (up, 4T1 p<0.0001, B16F10 p=0.0066; down, 4T1 p=0.0037, B16F10 p=0.0025) using two-sided Mann-Whitney test are represented. Number of TCs analyzed are indicated on the violin plots, 2 independent experiments were performed. **(C)** Single TCs volume at 15mpi (up) and 2hpi (down) of cells. Mean ± SD of TCs volume is shown and analyzed using two-sided Mann-Whitney test, 4T1 2hpi p=0.0047. Number of TCs analyzed indicated on the violin plots, 1 experiment was performed. **(D)** Representative images and bar graph quantification (% of nucleated cells) of lung-arrested viable 4T1 and B16F10 cells in the presence (black) or absence (red) of platelets. Scale: 10µm. Mean percentage ± SD of nucleated cells (number of TCs is indicated bove the histogram) are represented and analyzed using Kruskal-Wallis test following by the FDR method of Benjamini and Hochberg post-test, 2hpi q=0.0025 and q=0.0016 (1 experiment was performed). **(E)** Right: Representative confocal images of intravascular B16F10 clusters at 2hpi. Scale:10µm. Left: Violin plots showing the volume of platelet aggregates (AMIRA segmentation) around viable (V) and non-viable (NV) B16F10 at 15 mpi (p=0.004) and 2phi (p=0.022) and analyzed using two sided Mann-Whitney test (1 experiment was performed). **(F)** Left: Infographics showing the experimental setting for the TCs-platelets cluster formation *in vitro*. Right: Representative contrast phase microscope images of 4T1 and B16F10 cells in presence or absence of platelets. Scale bar: 10µm **(G)** Quantification of the ratio of clusters per single cells in presence or absence of platelets. Mean ratio of clusters per single cells (10 field of view in each group, indicated on the dot plots) is represented ± SD and analyzed using two-sided t

test, p=0.0093. **(H)** Quantification of the cluster area in presence or absence of platelets. Mean cluster area (pixel) is represented ± SD and analyzed using two-sided Mann-Whitney test, $p<0.0001$. Number of clusters analyzed is indicated on the dot plot. **(I)** Percentage of Zombie NIR+ cells as quantified by flow-cytometry measurement at 24 and 48 hours post clusters formation in presence or absence of platelets. Mean percentage of Zombie NIR^{+} cells is represented ± SD and analyzed using a two-sided t test, 1 experiment with 3 replicate was performed (20,000 cells per n), 4T1, 24h p=0.001 and 48h p=0.032, B16F10, 48h p=0.014. Source data are provided as a Source Data file.

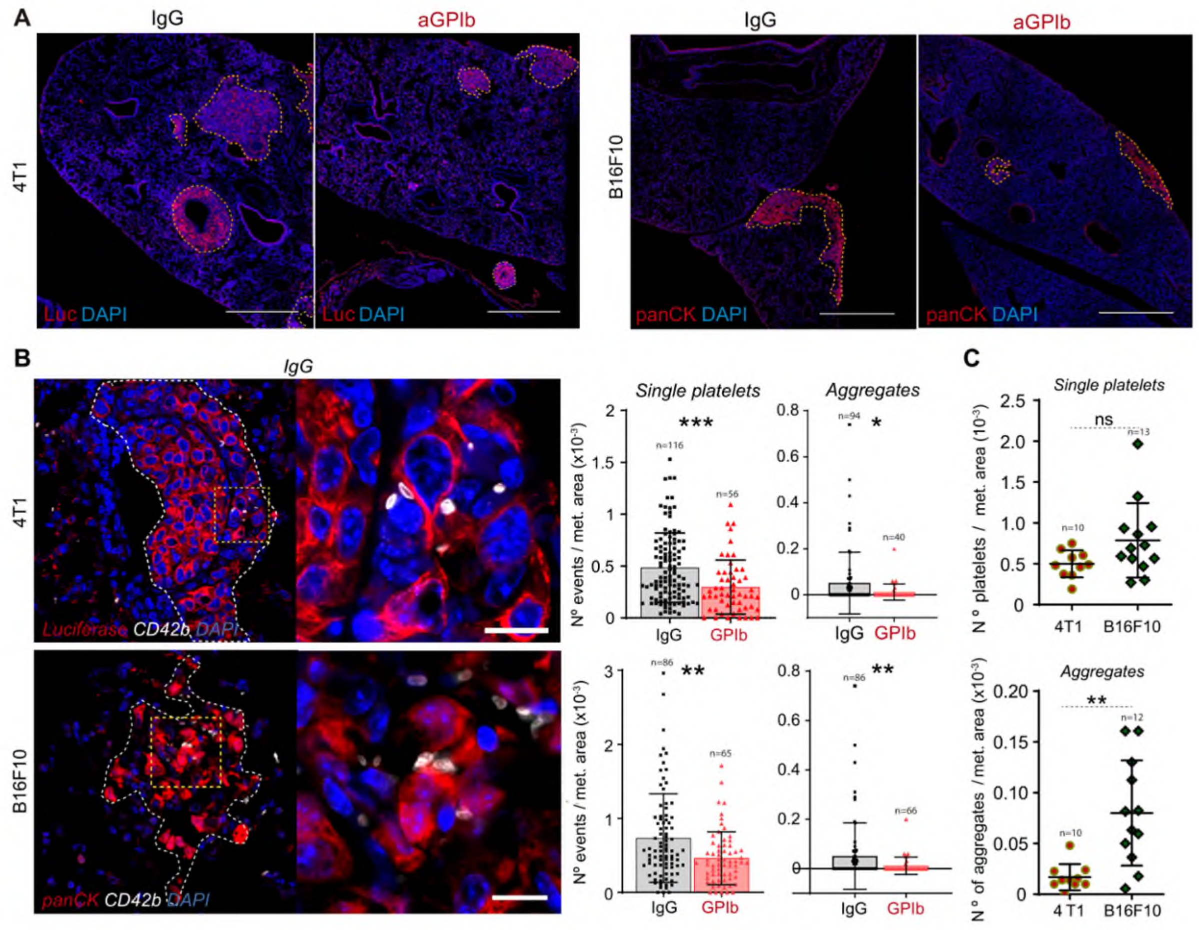

Supplementary Figure 4 (related to Figure 3). Garcia-Leon *et al.*

Supplementary Figure 4 *(related to Figure 3)*. ShortTCP treatment reduce metastatic burden and tackles platelets population and aggregation in metastatic foci (A) Low magnification confocal images stitches of lung metastases defined by luciferase (4T1) or pancytokeratin (B16F10) staining at 14dpi of control, short-term TCP mouse models injected with both 4T1 (left) and B16F10 (right) TCs. Scale bar: 500µm. **(B)** Left: Representative confocal images of mouse lung metastatic foci from 4T1 (upper) and B16F10 (bottom) cells at 14dpi stained for CD42b. Scale: 50µm. Right: number of single platelet events (≤ 3 platelets, left) and number of platelet aggregates (> 3 platelets together, right) per metastatic area (total number of areas analyzed are indicated in the histogram), defined by α-luciferase (4T1, top) or α-pancytokeratin staining (B16F10, bottom). Mean number of events (single platelets or aggregates) per metastatic area is represented as mean ± SD and analyzed using two-sided Mann-Whitney test, 4T1 single p=0.0003, aggregates p=0.0296; B16F10 single p=0.0034, aggregates p=0.0028. 3 independent experiments were performed. **(C)** Number of single platelets (top) and platelet aggregates (bottom) per metastatic area

(total number of areas analyzed are indicated on the dot plot) in IgG-treated animals for 4T1 and B16F10 models. Mean number of platelets (up) or aggregates (down) per metastatic area is represented as mean ± SD and analyzed using two-sided Mann-Whitney test, single p=0.0883, aggregates p=0.0011 (3 independent experiments were performed). Source data are provided as a Source Data file.

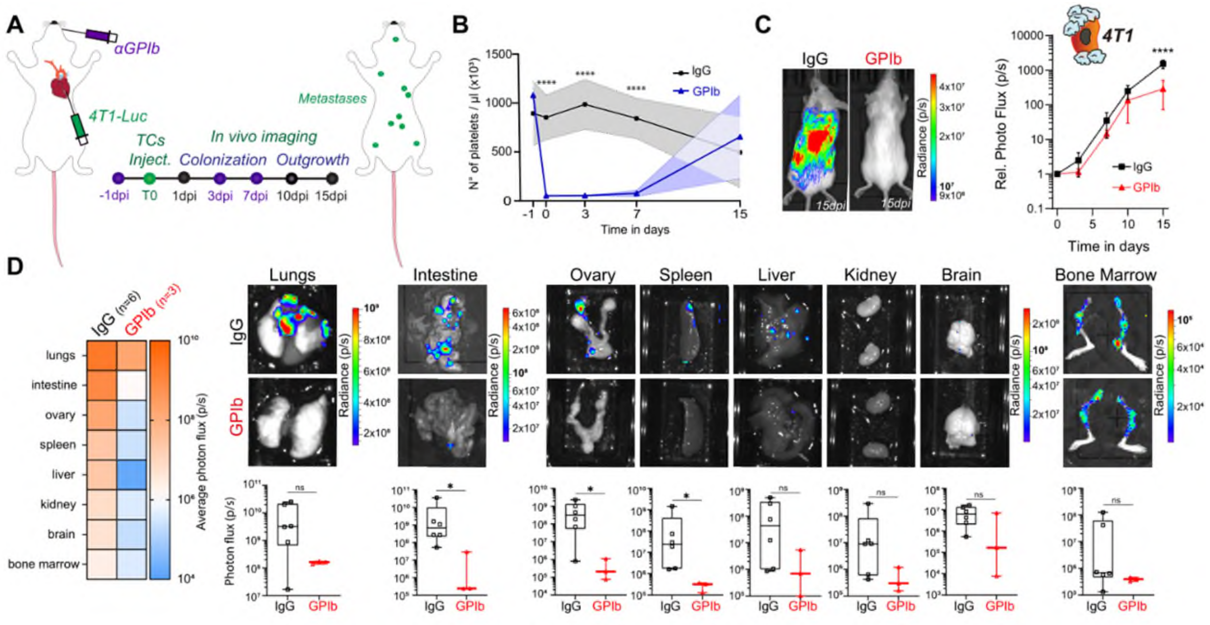

Supplementary Figure 5 (related to Figure 4). Garcia-Leon *et al.*

Supplementary Figure 5 (related to figure 4). Platelets' depletion affects also the pan-organ spreading of metastasis presents a pan-organs phenotype. **(A)** Infographics showing the experimental setting. **(B)** Graph showing platelet counts on IgG- and α-GPIb-treated Balb/c animals. Mean number of platelets per µL is represented ± SD and analyzed using two-way Anova with false discovery rate post-test, from 6 mice (IgG) and 3 mice (αGPIb) respectively, 1 independent experiment was performed. **(C)** Left: Representative images at 14dpi of total lung bioluminescence in IgG- versus αGPIb-treated animals. Right: Bioluminescence kinetics of 4T1-Luc TCs lung outgrowth in IgG- and α-GPIb-treated mice. Mean Relative photon flux is represented ± SEM and analyzed using two-way Anova with false discovery rate post-test, from 6 mice (IgG) and 3 mice (αGPIb) respectively, 1 independent experiment was performed, p=0.0003. **(D)** Left: Heatmap representing the Log10 of the *ex-vivo* photon flux of organs (average). Right: Representative images and quantification of *ex-vivo* photon flux of selected organs. Data are presented as min-max box-plot showing 25-75 percentile and median value and were analyzed using two-sided Mann-Whitney test from (intestine p=0.0238, ovary p=0.0476, spleen p=0.0238). Whiskers extend from the box to the farthest data points in both directions. Each point represents the value of a single mouse (n=6 IgG, n=3 αGPIb, 1 experiment was performed). Source data are provided as a Source Data file.

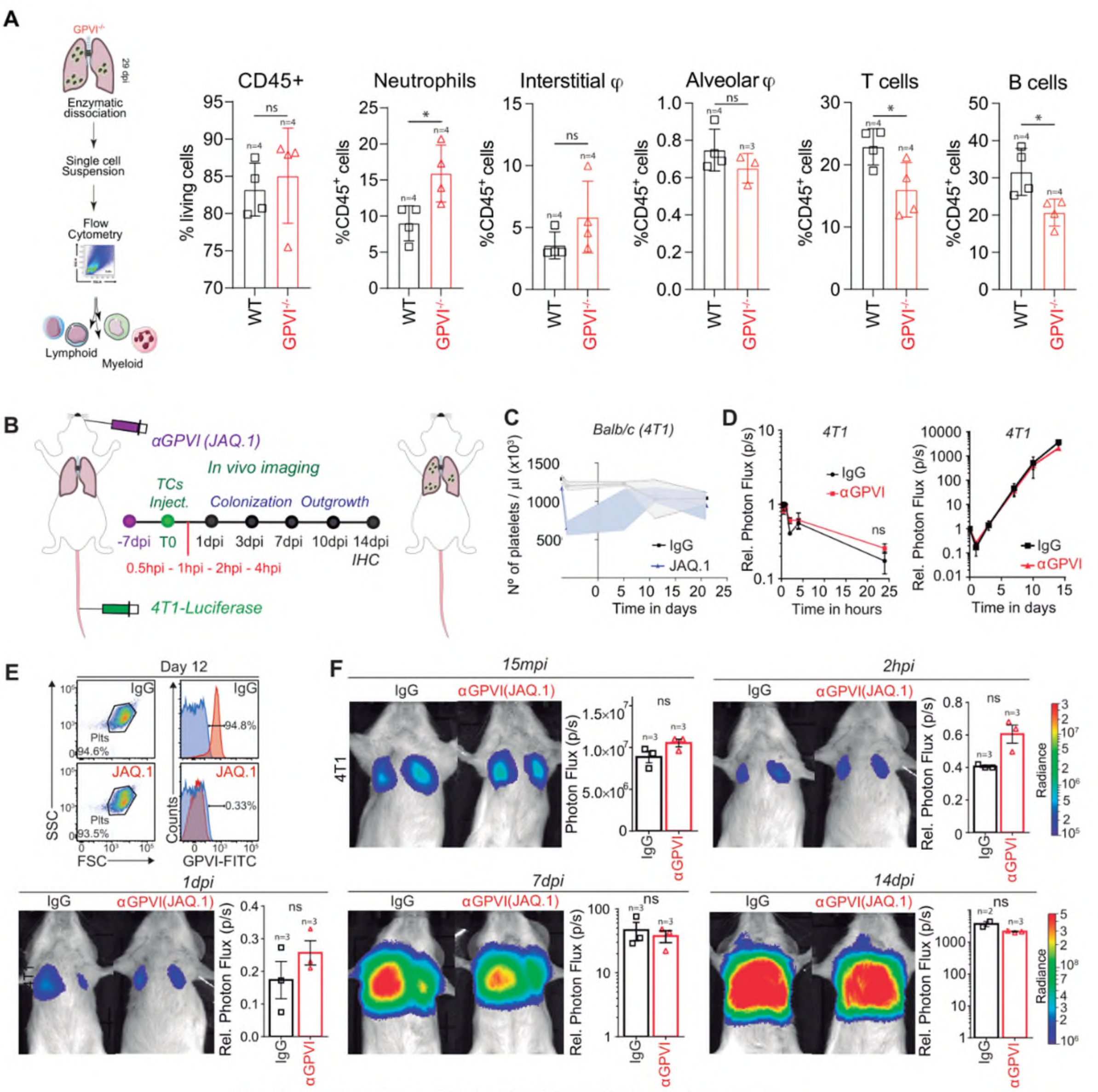

Supplementary Figure 6 (related to Figure 6). Garcia-Leon *et al.*

Supplementary Figure 6 *(related to Figure 6)*. GPVI differentially affects immune compartments in late timepoints (29dpi) and its targeting does not affect 4T1 cells metastatic outgrowth.

(A) *Ex-vivo* lungs immunophenotyping. Left: Infographics describing the experimental scheme. Right: percentage of relative immune cells populations as assessed by flow cytometry analysis 29dpi. Mean percentage of cell population (gated as indicated) is represented ± SD and analyzed using two-sided t test (neutrophils p=0.0249, T cells p=0.0388, B cells p=0.0244). Number of mice used per experiment indicated on the histograms (1 experiment was performed). **(B)** Infographics showing the experimental setting. **(C)** Platelets counts in normal vs JAQ.1-treated animals. Mean number of

platelets per µL is represented ± SD and analyzed using two-way Anova with false discovery rate post-test from n=3 mice, 1 experiment was performed. **(D)** Bioluminescence kinetics of 4T1-luc TCs lung seeding (left) and outgrowth (right) in normal and α-GPVI-depleted mice. Mean Relative photon flux is represented ± SEM and analyzed using two-way Anova with false discovery rate post-test from n=3 mice, 1 experiment was performed. **(E)** Flow cytometry gating of GPVI platelets' staining at 12dpi. Isotype in blue and αGPVI (JAQ.1) in red. **(F)** Representative images and time point (15mpi to 14dpi) comparisons of total lung bioluminescence in IgG versus GPVI-depleted animals. Mean photon flux or relative photon flux is represented ± SD and analyzed using two sided Mann-Whitney test. 3 mice per group, 1 experiment was performed. Source data are provided as a Source Data file.

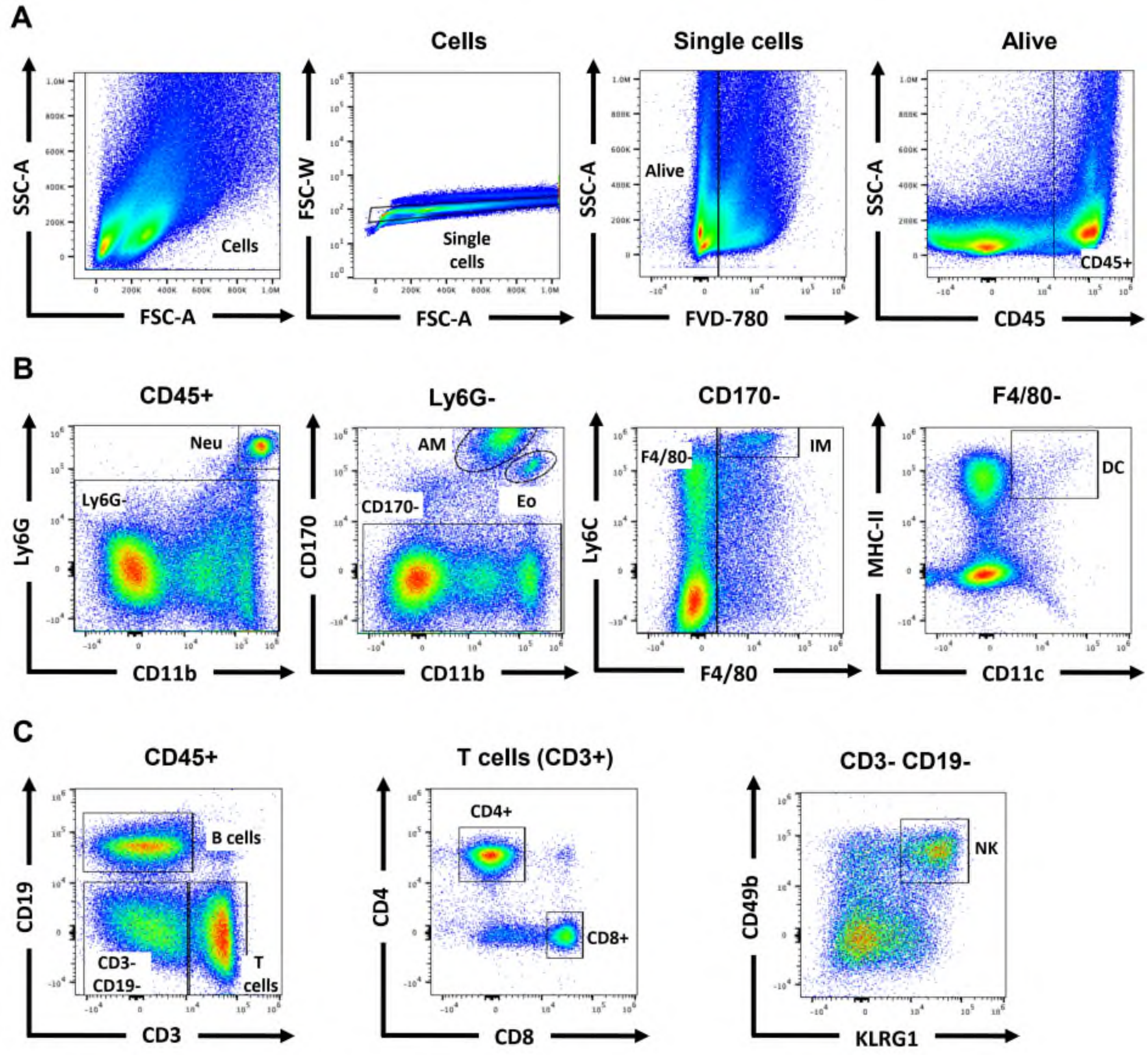

Supplementary Figure 7 ***(related to Figure 6)*****. Lung immunophenotyping gating strategy.**

Ex-vivo lungs immunophenotyping. **(A)** From left to right: gating strategy for hematopoietic inside the single cells and living cells gates (Fixable Viability Dye eFluor780 negative). **(B)** Gating strategy for the myeloid panel. From left to right: $CD45^+$ cells were first distinct in $Ly6G^+$ $CD11b^+$ neutrophils (Neu). Then, $CD170^+$ $CD11b^{med}$ alveolar macrophages (AM) and $CD170^{med}$ $CD11b^+$ eosinophils (Eo) were gated inside the $Ly6G^-$ cells. Further $Ly6G^-$ $CD170^-$ cells were distinguished in $F4/80^+$ interstitial macrophages (IM) and in $F4/80^+$ $MHC\text{-}II^+$ $CD11c^+$ dendritic cells (DC). **(C)** Gating strategy for the lymphoid panel. $CD45^+$ cells were divided in $CD19^+$ B cells, $CD3^+$ pan T cells and $CD19^-$ $CD3^-$ double negative cells. Pan T cells were further

distinguished in $CD4^+$/$CD8^+$ cells and $CD49b^+$ $KLRG1^+$ NK cells were found in the $CD19^-$ $CD3^-$ double negative cells.

Antibody conjugate	Clone or Ref.#	Supplier	Application
AF488 goat anti-rat (H+L)	#A11006	ThermoFisher	IF
AF555 goat anti-mouse (H+L)	#A21424	ThermoFisher	IF
AF555 goat anti-rabbit (H+L)	#A21429	ThermoFisher	IF
AF647 goat anti-rat (H+L)	#A21247	ThermoFisher	IF
Anti-firefly luciferase purified	#ab21176	Abcam	IF - Tumor cells
CD11b-PerCP/Cy5.5	M1/70	BioLegend	Lung immunophenotyping - myeloid cells
CD11c-BV605	N418	BioLegend	Lung immunophenotyping - myeloid cells
CD170 (SiglecF)-PE	S17007L	BioLegend	Lung immunophenotyping - myeloid cells
CD19-BV711	6D5	BioLegend	Lung immunophenotyping - B cells
CD25-BV421	A18246A	BioLegend	Lung immunophenotyping - T cells
CD3e-PerCP/Cy5.5	17A2	BioLegend	Lung immunophenotyping - T cells
CD41 purified	MWReg30	abcam	IF- Human Platelets
CD42c (GPIbβ) purified	RAM.1	In house (EFS)	IF - Murine Platelets
CD42c (GPIbβ) purified	RAM.6	In house (EFS)	In vivo platelets depletion
CD45-biotin	30-F11	BioLegend	IF - Immune cells
CD45-BV510	30-F11	BioLegend	Lung immunophenotyping
CD4-AF700	RM4-5	BioLegend	Lung immunophenotyping - T cells
CD8a-BV605	53-6.7	BioLegend	Lung immunophenotyping - T cells
Cytokeratin pan-mixture purified	#C2562	Sigma	IF - Tumor cells
DAPI	#00-4959-52	ThermoFisher	IF - Nucleus
F4/80-PE/Cy7	BM8	BioLegend	Lung immunophenotyping - macrophages
GPVI purified		Acticorp MTA	In vivo platelets binding competition
Fixable Viability Dye eFluor780	#65-0865-18	ThermoFisher	Lung immunophenotyping - Viability
I-A/I-E (MHC-II)-AF700	M5/114.15.2	BioLegend	Lung immunophenotyping - APCs
Ki67 purified	SP6	ThermoFisher	IF - Proliferation
KLRG-PE/Cy7	2F1/KLRG1	BioLegend	Lung immunophenotyping - macrophages
Ly6C-AF488	HK1.4	BioLegend	Lung immunophenotyping - myeloid cells
Ly6G-BV711	1A8	BioLegend	Lung immunophenotyping - myeloid cells
NG2	9.2.27	BD Pharmingen	IF-Human cells
NKp46-PE	29A1.4	BioLegend	Lung immunophenotyping - NK cells
PD-1-PE/Dazzle	29F.1A12	BioLegend	Lung immunophenotyping - T cells
Streptavidin-647	#405235	BioLegend	IF-Immune cells

Table S1. Antibodies list. List of antibodies used in immunofluorescence and flow-cytometry applications.
