# Supplementary material for: Platelets favor the outgrowth of established metastases
Source: Nat Commun. 2024 May 13;15:3297. doi: 10.1038/s41467-024-47516-w (PMC11091169; doi:10.1038/s41467-024-47516-w)
Supplement: Supplementary file 3 — Reporting Summary [file 41467_2024_47516_MOESM3_ESM.pdf]

nature portfolio

NCOMMS-23-45035A
Corresponding author(s): Maria-Jesus Garcia-Leon, Jacky G Goetz, Pierre H Mangin
Last updated by author(s): Feb 16, 2024

Reporting Summary

Nature Portfolio wishes to improve the reproducibility of the work that we publish. This form provides structure for consistency and transparency in reporting. For further information on Nature Portfolio policies, see our Editorial Policies and the Editorial Policy Checklist.

Statistics

For all statistical analyses, confirm that the following items are present in the figure legend, table legend, main text, or Methods section.

n/a	Confirmed	
☐	☒	The exact sample size (*n*) for each experimental group/condition, given as a discrete number and unit of measurement
☐	☒	A statement on whether measurements were taken from distinct samples or whether the same sample was measured repeatedly
☐	☒	The statistical test(s) used AND whether they are one- or two-sided<br>*Only common tests should be described solely by name; describe more complex techniques in the Methods section.*
☒	☐	A description of all covariates tested
☐	☒	A description of any assumptions or corrections, such as tests of normality and adjustment for multiple comparisons
☐	☒	A full description of the statistical parameters including central tendency (e.g. means) or other basic estimates (e.g. regression coefficient) AND variation (e.g. standard deviation) or associated estimates of uncertainty (e.g. confidence intervals)
☐	☒	For null hypothesis testing, the test statistic (e.g. F, t, r) with confidence intervals, effect sizes, degrees of freedom and P value noted<br>*Give P values as exact values whenever suitable.*
☒	☐	For Bayesian analysis, information on the choice of priors and Markov chain Monte Carlo settings
☒	☐	For hierarchical and complex designs, identification of the appropriate level for tests and full reporting of outcomes
☒	☐	Estimates of effect sizes (e.g. Cohen's *d*, Pearson's *r*), indicating how they were calculated

Our web collection on statistics for biologists contains articles on many of the points above.

Software and code

Policy information about availability of computer code

Data collection	Data were collected using Microsoft Excel 2013 (Windows 10), Graph Pad Prism 5 and 9, IVIS Lumina III and Living Image Software 4.7 (Perkin Elmer), FACS Diva 9.0.1 (BD Biosciences), Scil Vet abc automatic cell counter (Scil Animal Care Company), automatic platelet counter XN-1000 (Sysmex), APACT 4004 aggregometer and APACT LPC Software (ELITech Group), Phenom-World SEM desktop microscope (Phenon-World B.V.), TEM Hitachi 7500 with a Hamamatsu camera C4742-51-12NR, Leica TCS SP8 laser scan confocal microscope using a 20x/1.0 W-Plan-Apochromat objective (NA 0.75) and a 63xPlan-Apochromat (NA 1.4) and a Leica M205 FA stereomicroscope.
Data analysis	Data were analyzed using Microsoft Excel 2013 (Windows 10), Graph Pad Prism 5 and 9, Living Image Software 4.7 (Perkin Elmer), Matlab 2016, FACS Diva 9.0.1 (BD Biosciences), FlowJo 10 (TreeStar), AMIRA Visage 5.3.3, IMARIS 9.5.1, Adobe Photoshop CS4 and ImageJ 1.53f51 (FIJI).

For manuscripts utilizing custom algorithms or software that are central to the research but not yet described in published literature, software must be made available to editors and reviewers. We strongly encourage code deposition in a community repository (e.g. GitHub). See the Nature Portfolio guidelines for submitting code & software for further information.

Data

Policy information about availability of data

All manuscripts must include a data availability statement. This statement should provide the following information, where applicable:

- Accession codes, unique identifiers, or web links for publicly available datasets
- A description of any restrictions on data availability
- For clinical datasets or third party data, please ensure that the statement adheres to our policy

All data supporting the findings of this study are available from the corresponding authors on reasonable request. Data generated by FFPE RNA-Seq have been deposited in public databases.

Research involving human participants, their data, or biological material

Policy information about studies with human participants or human data. See also policy information about sex, gender (identity/presentation), and sexual orientation and race, ethnicity and racism.

Reporting on sex and gender	All human specimens derived from women.
Reporting on race, ethnicity, or other socially relevant groupings	Unknown
Population characteristics	Female patients diagnosed with metastatic melanoma. Samples were collected from lung biopsies of metastatic sites.
Recruitment	The recruitment criteria was metastatic melanoma diagnosis confirmed by anatomic pathology.
Ethics oversight	Human studies were performed according to Helsinki declaration and regulations provided by the Ethics Commitee from Hospital Gregorio Maranon, Madrid, Spain, Protocol Version 05/Junio 2018. Control human blood samples were obtained from volunteer blood donors who gave written informed consent recruited by the blood transfusion center where the research was performed (Etablissement Français du Sang, Grand-Est). All procedures were registered and approved by the French Ministry of Higher Education and Research. The donors gave their approval in the CODHECO number AC- 2008 - 562 consent form, for the samples to be used for research purposes.

Note that full information on the approval of the study protocol must also be provided in the manuscript.

Field-specific reporting

Please select the one below that is the best fit for your research. If you are not sure, read the appropriate sections before making your selection.

☒ Life sciences ☐ Behavioural & social sciences ☐ Ecological, evolutionary & environmental sciences

For a reference copy of the document with all sections, see nature.com/documents/nr-reporting-summary-flat.pdf

Life sciences study design

All studies must disclose on these points even when the disclosure is negative.

Sample size	Sample size was determined by pilot studies that have given statistically significant results and literature search. In the case of mouse experimentation the 3R ethical guidelines were applied after confirmation of low variability between individuals in pilot studies. Sample size for human data were selected based upon the availability of patient biopsies of lung metastatic cancer.
Data exclusions	Less than 5 individual data points within the whole manuscript's datasets were excluded from analysis after their proper statistical identification as outlayers using the Grubbs' test, also called the ESD method (extreme studentized deviate), to determine whether the are significant outliers from the rest. Alpha value was set at 0.05.
Replication	A different number of replicates were done depending on the type of experiment. For in vitro experiment from N=3 to 7 replicates were done, depending on the variability of the results and the relevance of the model (subsequent use at in vivo assays). For consistent results in non-relevant cell lines we established N=3 as a maximum number of triplicates, for the cell models chosen as working models, we went up to N=5, 6 or 7.<br>In the case of in vivo experiment, the number of replicates was reduced but also depending on the type of in vivo model. For zebrafish experiments, we have replicated the assays from N=3 to N=4 depending on the availability of the animal model. For mouse experiments, replicates were diminished up to N=2 according to the 3Rs ethical guidelines, after initial confirmation of low variability between individual results in pilot experiments. Number of replicates of each experiment is indicated in the corresponding figure legend.
Randomization	Animals were randomly allocated into all the experimental groups.
Blinding	Investigators were not blinded during data collection and/or analysis but experiments were performed by two different individuals. Blinding

Blinding	was not recommended in mouse experiments involving thrombocytopenia, as thrombocytopenic subjects needed to be manipulated carefully to avoid premature dead during experimentation due to internal bleeding.

Reporting for specific materials, systems and methods

We require information from authors about some types of materials, experimental systems and methods used in many studies. Here, indicate whether each material, system or method listed is relevant to your study. If you are not sure if a list item applies to your research, read the appropriate section before selecting a response.

Materials & experimental systems

n/a	Involved in the study	
☐	☒	Antibodies
☐	☒	Eukaryotic cell lines
☒	☐	Palaeontology and archaeology
☐	☒	Animals and other organisms
☒	☐	Clinical data
☒	☐	Dual use research of concern
☒	☐	Plants

Methods

n/a	Involved in the study	
☒	☐	ChIP-seq
☐	☒	Flow cytometry
☒	☐	MRI-based neuroimaging

Antibodies

Antibodies used

Anti-mouse GPIb, clone RAM.1, in-house produced, Etablisment Francaise du sang (EFS) (Strassel, C. et al. 2006).
Anti-mouse GPIb, clone RAM.6, in-house produced, Etablisment Francaise du sang (EFS) (Strassel, C. et al. 2006).
Anti-mouse purified NA/LE Rat IgG2a, κ Isotype Control, BD Biosciences, catalog number: 555840.
Anti-mouse GPVI Rat (Wistar) IgG2a, clone JAQ.1, Emfret Analytics, Catalog number: M011-0.
Anti-human GPVI, Acticorp cession under MTA agreement.
Anti-mouse Ki-67 Monoclonal Antibody IgG, Unconjugated, clone SP6, Thermo Fischer, catalog number: MA5-14520 and RM-14, catalog number replacement. Company validated plus numerous citations.
Anti-mouse CD45 Biotinylated, clone 30-F11, Biolegend, catalog number: 103104. Company validated plus numerous references.
Anti-mouse-Cytokeratin, monoclonal pan (Mixture IgG1, IgG2a), clones C-11 + PCK-26 + CY90 + KS + 1A3 + M20 + A53-B/A2.
Anti-firefly Luciferase, polyclonal IgG, Abcam, catalog number ab21176.
Anti-mouse purified GPIb⍰ (CD42b), monoclonal Rat (Wistar) IgG, clone Xia.G7, Emfret Analytics, catalog number: M042-0.
Anti-rabbit IgG (H+L), highly cross-absorbed polyclonal, Alexa Fluor-555 coupled, Thermo Fisher, catalog number: A-21429.
Anti-mouse IgG (H+L), highly cross-absorbed polyclonal, Alexa Fluor-555 coupled, Thermo Fisher, catalog number: A-21424.
Anti-mouse CD11b-PerCP/Cy5.5 , clone : M1/70, BioLegend
Anti-mouse CD11c-BV605, clone: N418, BioLegend
Anti-mouse CD170 (SiglecF)-PE , clone: S17007L , BioLegend
Anti-mouse CD19-BV711, clone: 6D5, BioLegend
Anti-mouse CD25-BV421 , clone: A18246A, BioLegend
Anti-mouse CD3e-PerCP/Cy5.5 , clone: 17A2, BioLegend
Anti-mouse CD45-BV510 , clone: 30-F11, BioLegend
Anti-mouse CD4-AF700, clone: RM4-5, BioLegend
Anti-mouse CD8a-BV605, clone: 53-6.7, BioLegend
Anti-mouse F4/80-PE/Cy7, clone: BM8, BioLegend
Fixable Viability Dye eFluor780, catalogue #65-0865-18 , ThermoFisher
Anti-mouse I-A/I-E (MHC-II)-AF700 , clone: M5/114.15.2, BioLegend
Anti-mouse KLRG-PE/Cy7, clone: 2F1/KLRG1, BioLegend
Anti-mouse Ly6C-AF488, clone: HK1.4, BioLegend
Anti-mouse Ly6G-BV711, clone: 1A8 , BioLegend
Anti-mouse NKp46-PE , clone:29A1.4, BioLegend
Anti-mouse PD-1-PE/Dazzle, clone: 29F.1A12, BioLegend
Anti human NG-2 clone: 9.2.27, BD Pharmingen

Validation

In-house RAM1 anti-mouse platelet GPIb antibody was validated for in vivo use here: Perrault C. et al. 2001. Throm. Haemost.
In-house RAM6 anti-mouse platelet GPIb antibody was validated for in vivo use here: Strassel, C. et al. 2006. Journal of thrombosis and hemostasis. This antibody is equivalent to the following commercial version used in pilot studies: https://www.emfret.com/fileadmin/user_upload/Datasheets/R300.pdf
Anti-mouse GPVI, clone JAQ.1 was previously validated for in vivo use here: Nieswandt B. et al. 2018. J. Exp. Med.
Anti-human GPVI (Glenzocimab) was validated for in vivo use here: Jadoui S. et al. 200—2003. Haematologica.
Anti-mouse purified NA/LE Rat IgG2a Isotype Control, BD Biosciences was validated for in vivo use by the providing company. https://www.bdbiosciences.com/eu/reagents/research/antibodies-buffers/immunology-reagents/anti-human-antibodies/cell-surface-antigens/purified-nale-rat-igg2a-isotype-control-r35-95/p/555840
Anti-mouse CD45 Biotinylated, clone 30-F11 has been validated by the providing company and by numerous references https://www.biolegend.com/fr-fr/products/biotin-anti-mouse-cd45-antibody-98
Anti-mouse Ki-67, clone SP6, has been validated by the providing company and by numerous references. https://www.thermofisher.com/antibody/product/Ki-67-Antibody-clone-SP6-Recombinant-Monoclonal/MA5-14520
Anti-mouse-Cytokeratin, monoclonal pan (Mixture IgG1, IgG2a), has been validated by the providing company and by numerous references. http://www.sigmaaldrich.com/catalog/product/sigma/c2562?lang=en&region=US, and also by first author previous work: Garcia-Leon M.J. et al. 2018. Development.

Anti-firefly Luciferase has been validated by the providing company and by numerous references. http://www.abcam.com/firefly-luciferase-antibody-ab21176.html
Anti-mouse purified GPIb (CD42b), clone Xia.G7 was validated by the providing company for the detection of platelets by flow cytometry and by pilot studies on tissue OCT and paraffin slides. https://fnkprddata.blob.core.windows.net/domestic/data/datasheet/EMF/M042-1.pdf
Anti NG-2 antibody has been advanced validated by the providing company https://www.thermofisher.com/antibody/product/Neural-Glial-Antigen-2-NG2-Antibody-clone-9-2-27-Monoclonal/14-6504-82
All immunofluorescence antibodies were further validated in the laboratory using negative staining controls.
CD11b-PerCP/Cy5.5 clone M1/70 was validated by the company and it is referenced in 201 publications https://www.biolegend.com/en-us/products/purified-anti-mouse-human-cd11b-antibody-351
CD11c-BV605 clone N418 was validated by the company and it is referenced in 135 publications https://www.biolegend.com/en-us/products/purified-anti-mouse-cd11c-antibody-1817
CD170 (SiglecF)-PE clone S17007L was validated by the company and it is referenced in 2 publications https://www.biolegend.com/en-us/products/purified-anti-mouse-cd170-siglec-f-antibody-16369
CD19-BV711 clone 6D5 was validated by the company and it is referenced in 61 publications https://www.biolegend.com/en-us/products/purified-anti-mouse-cd19-antibody-1532
CD25-BV421 clone A18246A was validated by the company https://www.biolegend.com/en-us/products/purified-anti-mouse-cd25-antibody-22049
CD3e-PerCP/Cy5.5. clone 17A2 was validated by the company and it is referenced in 111 publications https://www.biolegend.com/en-us/products/purified-anti-mouse-cd3-antibody-48
CD4-AF700 clone RM4-5 was validated by the company and it is referenced in 46 publications https://www.biolegend.com/en-us/products/alexa-fluor-700-anti-mouse-cd4-antibody-3386
CD8a-BV605 clone 53-6.7 was validated by the company and it is referenced in 74 publications https://www.biolegend.com/en-us/products/brilliant-violet-605-anti-mouse-cd8a-antibody-7636
F4/80-PE/Cy7 clone BM8 was validated by the company and it is referenced in 206 publications https://www.biolegend.com/en-us/products/pe-cyanine7-anti-mouse-f4-80-antibody-4070
I-A/I-E (MHC-II)-AF700 clone M5/114.15.2 was validated by the company and it is referenced in 106 publications https://www.biolegend.com/en-us/products/alexa-fluor-700-anti-mouse-i-a-i-e-antibody-3413
KLRG-PE/Cy7 clone 2F1/KLRG1 was validated by the company and it is referenced in 31 publications https://www.biolegend.com/en-us/products/pe-cyanine7-anti-mouse-human-klrg1-mafa-antibody-8312
Ly6C-AF488 clone HK1.4 was validated by the company and it is referenced in 42 publications https://www.biolegend.com/en-us/products/alexa-fluor-488-anti-mouse-ly-6c-antibody-6756
Ly6G-BV711 clone 1A8 was validated by the company and it is referenced in 14 publications https://www.biolegend.com/en-us/products/brilliant-violet-711-anti-mouse-ly-6g-antibody-12062
NKp46-PE clone 29A1.4 was validated by the company and it is referenced in 30 publications https://www.biolegend.com/en-us/products/pe-anti-mouse-cd335-nkp46-antibody-6523
PD-1-PE/Dazzle clone 29F.1A12 was validated by the company and it is referenced in 8 publications https://www.biolegend.com/en-us/products/pe-dazzle-594-anti-mouse-cd279-pd-1-antibody-12090
All flow-cytometry antibodies were also internally validated by negative staining and single stainings.
Secondary antibodies are routinely used in the lab for many applications and validated by the provider (Thermo Fisher)

Eukaryotic cell lines

Policy information about cell lines and Sex and Gender in Research

Cell line source(s)

B16F10 cells were purchased at Perkin Elmer. MDA-MB-231 and MCF7 cell lines were purchased from ATCC. EO771 cells were purchased from Ch3 biosystems. AT3 were a kind gift from Abrams S.I. Lab: Stewart T.J. 2007. J. Immunol. 4T1 cells were a kind gift from Corine Laplace-Builhé (Institut Gustave Roussy). D2A1 were a kind gift from Bob Weinberg lab. Finally, A375 cells where gifted by Guillaume Montagnac.

Authentication

Consistency in cell line phenotypes was ensured in all cases by the application of the seed-loot system for cell banking, where master and working stocks were generated from an initial (commercial, or not) vial. Experiments were performed with low-passage working stock vials.
B16F10 cell line was authenticated by the provider company (Perkin Elmer). We validated their epithelial phenotype by expression and secretion to the culture media of melanin and their non-epithelial phenotype by staining with anti-pan-cytokeratin antibody (negative). Proper doubling time was also studied according to ATCC cell line description.
4T1 cells were previously used in Ghoroghi S. et al. 2021. eLife, and many other papers from the lab. They are also a well-established model of breast cancer metastasis in the literature. We validated their epithelial phenotype by immunostaining with pan-cytokeratin. Their exponential growth in Balb/c mice confirmed their genetic background. Proper doubling time was also studied as indicated at ATCC cell line description.
E0771 cell line was authenticated by the provider company (Ch3 biosystems). Proper doubling time was studied according to the company's cell line description.
The rest of the cell lines, used only for in vitro tests, were validated by assessing doubling time and mycoplasma negativity.

Mycoplasma contamination

All cell lines used were mycoplasma negative. Tested with the Venor GeMOneStep Mycoplasma detection kit, Thermo Fisher.

Commonly misidentified lines
(See ICLAC register)

Non commonly misidentified cell lines were used in this study.

Animals and other research organisms

Policy information about studies involving animals; ARRIVE guidelines recommended for reporting animal research, and Sex and Gender in Research

Laboratory animals	Mouse (mus musculus), BALB/c or C57BL/6 from 8 to 12 weeks old from Charles Rivers laboratories, and C5BL/6 GPVI-deficient mice and hGPVI transgenic mice were generated and breeded in-house at the Etablishment Francais du Sang, Alsace (EFS) (Mangin P. H. et al. 2012. The journal of pharmacology and experimental therapeutics). Tg(fli1a:eGFP) Zebrafish (Danio rerio) embryos from a Tubingen background were kindly provided by the group of F. Peri from EMBL (Heidelberg, Germany) and further grown and bred in our in-house Zebrafish facility. Embryos were maintained as previously described (Goetz et al., 2014 Cell Reports)
Wild animals	N/A
Reporting on sex	For breast cancer modeling only females were used. Otherwise male and females were used in equivalent proportions.
Field-collected samples	N/A
Ethics oversight	Immunocompetent and genetically-modified mice used in this study were housed under pathogen-free conditions and all procedures were performed in accordance with the European Union Guideline 2010/63/EU. The study was approved by the Regional Ethical Committee for Animal Experimentation of Strasbourg, CREMEAS (CEEA 35) and registered under the APAFIS authorization 14741-2018041816337540 and 37433-2022052016445806

Note that full information on the approval of the study protocol must also be provided in the manuscript.

Plants

Seed stocks	N/A
Novel plant genotypes	N/A
Authentication	N/A

Flow Cytometry

Plots

Confirm that:

☒ The axis labels state the marker and fluorochrome used (e.g. CD4-FITC).

☒ The axis scales are clearly visible. Include numbers along axes only for bottom left plot of group (a 'group' is an analysis of identical markers).

☒ All plots are contour plots with outliers or pseudocolor plots.

☒ A numerical value for number of cells or percentage (with statistics) is provided.

Methodology

Sample preparation	Mouse whole blood was collected and as detailed in Material and Methods. Platelets in whole blood were stained with an anti-platelet antibody in-house made (and referenced in Methods) directly coupled with alexa-647. For ex-vivo flow cytometry, lung left lobes were cut and dissociated enzimatically according to the protocol stated in Material and Methods section. Antibodies used are listed in Table S1.
Instrument	BD LSRFortessa™ X-20 Cell Analyzer and Attune NxT (Invitrogen)
Software	Acquisition software used was the BD FACS Diva 9.0.1; analysis was done on FlowJo V10.
Cell population abundance	No sorting was applied in the present study.
Gating strategy	FACS analysis was performed in PBS-diluted mouse whole blood. FSC-A/SSC-W were used to discern platelets from background and big mononuclear cells. To separate platelet signal from erythrocytes, we stained with the platelet-specific antibody anti-GPIb (RAM1), directly labeled with alexa-647 and did an electronic gating on GPIb+ events. In the resulting gate, the expression of the marker in study (GPVI) was analysed. For ex-vivo lung immunophenotyping, FSC-A/SSC-A were used to gate the total cell population, further restricted to single cells events (FSC-A vs FSC-W) and to viable cells (negative at

10^4 for viability marker). Subpopulations gatings were sequentially within the CD45-expressing cell populations, according to the gating strategy in supplementary information (S7).

☒ Tick this box to confirm that a figure exemplifying the gating strategy is provided in the Supplementary Information.
